# Effects of Glycerol and Sodium Pentaborate Formulation on Prevention of Postoperative Peritoneal Adhesion Formation

**DOI:** 10.1155/2020/3679585

**Published:** 2020-04-06

**Authors:** Erhan Aysan, Fikrettin Sahin, Ruzgar Catal, Mirkhaliq Javadov, Alev Cumbul

**Affiliations:** ^1^Yeditepe University, Faculty of Medicine, Department of General Surgery, Istanbul, Turkey; ^2^Yeditepe University, Faculty of Engineering, Department of Genetic and Bioengineering, Istanbul, Turkey; ^3^Yeditepe University, Medical School Student, Istanbul, Turkey; ^4^Yeditepe University, Department of Histology and Embriology, Istanbul, Turkey

## Abstract

**Background:**

Postoperative peritoneal adhesions (PPA) are a serious problem for abdominal surgery. An effective remedy has not been found yet. New formulation of glycerol and sodium pentaborate may be able to solve the problem.

**Method:**

Female Wistar albino rats were randomly assigned into four equal groups. The adhesion model was created on the caecum anterior wall and covered with 2 ml 0.9% NaCl, 3% glycerol, 3% sodium pentaborate, and 3% glycerol plus 3% sodium pentaborate solutions in the groups, respectively. Two weeks later, the rats were sacrificed. PPA were graded macroscopically and microscopically.

**Results:**

Total adhesion scores of the 3% glycerol + 3% sodium pentaborate group were statistically different from the other groups for macroscopic and also microscopic evaluations (*p* < 0.001).

**Conclusion:**

3% glycerol plus 3% sodium pentaborate as a new formulation has preventive effects on PPA with a synergistic mechanism.

## 1. Introduction

Postoperative peritoneal adhesion (PPA) is a serious problem in abdominal surgery. It is one of the most common causes of mechanical intestinal obstruction, female infertility, and pelvic pain [1–3]. PPA also causes serious health care expenditure [4]. Several products and techniques have been suggested to prevent PPA, but no effective remedy has been found.

Glycerol is a viscous liquid alcohol with a molecular weight of 92.09 daltons [3]. It dissolves in water and alcohols, but not in liquid hydrocarbons [5]. Glycerol is one of the most common molecules in living organisms, and it is also a central component of lipids. Fatty tissues consist of one molecule of glycerol combined with three molecules of fatty acids [6, 7]. Glycerol is used in medical, pharmaceutical, and personal care preparations, mainly as a means of improving smoothness, providing lubrication, and as a humectant (a hygroscopic substance) [6, 7]. In a study conducted by our group, PPA prevention was shown using 1% glycerol [8].

Boron is a very stable metal. Groundwater contains a small amount of boron. The richest sources of boron are fruits, vegetables, pulses, legumes, and nuts. The mean daily intake of boron in the diet is estimated to be about 1.2 mg/day. As boron is very similar to the carbon atom, many carbon-based molecules are the same as the boron-based molecules [1]. The wound healing benefits of some types of boron compounds, especially sodium pentaborate, have been previously documented [9–14].

In this research, we aimed to investigate the effects of 3% glycerol and 3% sodium pentaborate compound on PPA prevention. We hypothesized that the new formulation may be more beneficial for the prevention of PPA than 1% glycerol due to synergistic activity.

## 2. Method

The study protocol was approved by the local animal ethics committee. Twenty-eight female Wistar albino rats (mean weight 270 ± 35 g, mean age 6 months, out bred) were randomly assigned into four groups of 7 rats each, calculated to yield results with 0.9 power and 0.05 confidence interval.

Following overnight fasting, all animals were anesthetized by intramuscular 75 mg/kg ketamine. The mid-abdominal surfaces were shaved and prepared with povidone-iodine. For each rat, the peritoneal cavity was entered through a 2-cm midline incision, and the caecum was mobilized and the anterior surface was scraped with sterile dry gauze until serosal petechias appeared (scraping model) [1]. The created surfaces from the adhesion model were covered with 2  ml 0.9% NaCl, 3% glycerol, 3% sodium pentaborate, and 3% glycerol plus 3% sodium pentaborate solutions in the groups, respectively.

The abdominal incisions were closed in two layers with continuous 3/0 polypropylene sutures. The animals were then placed on the regular pellet (state manufacturer) food. Two weeks later, the rats were sacrificed with an overdose of ketamine, and the peritoneal cavities were entered with a reversed U-shaped incision of the anterior abdominal wall, which was retracted caudally to provide maximal exposure. Adhesions were graded as 0–3 according to size and severity scoring [15] (Table 1).

The injured caecum surfaces with adhesion formations were excised and put into formaldehyde solutions. The specimens were fixed in 70% alcohol, dehydrated, and embedded in paraffin wax. Sections were cut at a thickness of 5 mm, stained with hematoxylin and eosin, and evaluated according to histopathologic fibrosis scoring [16] (Table 2). Total adhesion scores were evaluated and compared for the groups. The total adhesion score was calculated by multiplying the number of animals and the adhesion grades.

### 2.1. Statistical Analysis

Statistical analyses were performed using GraphPad Prisma V.10 software. Results were evaluated with a confidence interval of 95% and *p* < 0.05. The Chi-square and Fisher tests were used for intergroup comparisons.

## 3. Results

**Figure 1:**
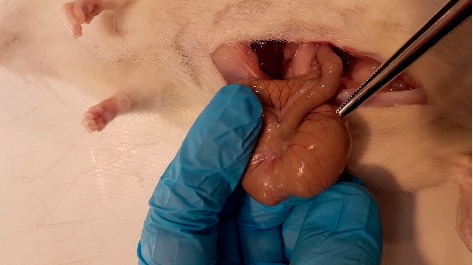
Macroscopic adhesion score 0 case from the 3% glycerol plus 3% sodium pentaborate group.

**Figure 2:**
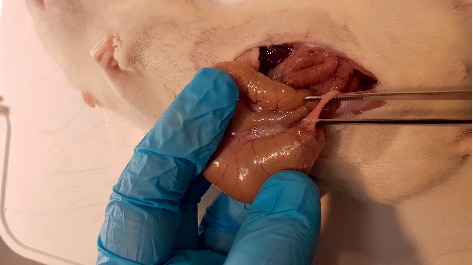
Macroscopic adhesion score 1 case from the 3% glycerol group.

**Figure 3:**
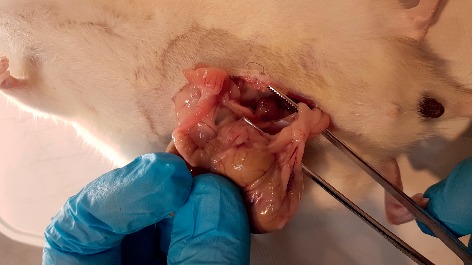
Macroscopic adhesion score 2 case from the 3% sodium pentaborate group.

**Figure 4:**
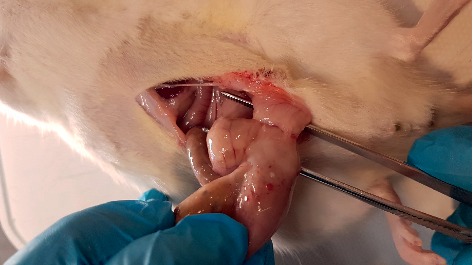
Macroscopic adhesion score 3 case from the control group.

Grades of macroscopic adhesion size and severity of the groups are shown in Tables 3 and 4. Grades of histopathologic fibrosis are shown in Table 5. Total adhesion scores of the 3% glycerol plus 3% sodium pentaborate group were statistically different from the other groups for macroscopic and also microscopic evaluations (*p* < 0.001, Figure 1). The total macroscopic adhesion size score was statistically different in the 3% glycerol group (*p* < 0.001, Figure 2) but not in the 3% sodium pentaborate group (*p* > 0.05, Figure 3) compared with the 0.9% NaCl group (Figure 4), respectively. The total macroscopic adhesion severity score was statistically different in the 3% glycerol group (*p* < 0.001) and also the 3% sodium pentaborate group (*p* < 0.05) compared with the 0.9% NaCl group respectively. Total histopathologic fibrosis scores of the 3% glycerol and the 3% sodium pentaborate groups were the same and statistically different (*p* < 0.05) form the 0.9% NaCl group.

## 4. Discussion

Trauma to living tissue causes a wound and triggers the start of the wound healing process. Wound healing occurs in three stages: inflammation, fibrosis, and maturation. Many proteins, molecules, cells, and cytokines are involved in the inflammation stage. The effect and mechanism of action of many of these have not been elucidated. Many molecules that emerge during inflammation have adhesive properties. The amount of adhesion formation is related to the severity and duration of the inflammation [17, 18]. In the second stage of wound healing, fibrinogen proteins turn into thin fibrin fibers. These fibers then combine to form thicker and stronger collagen fibers. The maturation stage is the continuation of the fibrosis stage, where collagen fibers are organized. Collagen fibers are characterized by forming strong bands (adhesions) to the surfaces that they contact regardless of whether they belong to the wound or not [17, 19].

Many peritoneal manipulations (holding, pulling, stretching, and cutting) cause a wound. After this, the peritoneal wound healing process starts and always leads to PPA formation [1, 2, 20–22]. Several techniques, substances, and agents have been investigated to prevent PPA. These include various surgical methods, minimal invasive and laparoscopic techniques, pharmacological agents targeting the inflammatory response and/or fibrin formation, liquids to form a mechanical barrier between mesothelial surfaces, gels and solids, etc. Although some techniques or agents have proven useful, none have shown complete success [1, 20–23].

Basic principles for PPA prevention are reduction of inflammation (anti-inflammatory effect), acceleration of wound healing, and separation of the wound surfaces from surrounding tissues. Although these principles are known, many studies have been conducted to prevent PPA, but an effective product has yet to be discovered [16, 24–26].

We have also studied on this topic previously [8] and revealed that 1% glycerol is effective for the prevention of PPA. According to macromolecular structure, when injected into the tissues or spaces into the body, absorption of glycerol via capillaries is difficult. So, glycerol stays in the injected area for a long period of time [5–7]. As glycerol is a very biocompatible molecule, it is used in many pharmaceutical, cosmetic, and prosthetic products [6, 7, 27–29]. Glycerol is frequently added to peritoneal dialysis solutions. Mortier et al. reported that glycerol augments the efficacy of peritoneal dialysis and ensures a protective effect on peritoneal surfaces [30]. In our previous study, we revealed that 1% glycerol was effective for the prevention of PPA by mechanically separating the peritoneal healing surfaces from surrounding tissues [8].

Mechanical separations using gelatinous liquids with high viscosity have yielded relatively high success rates. Gelatinous liquids are thought to prevent the formation of PPA by providing a protective layer between the surfaces and preventing contact between deperitonized surfaces and surrounding tissues [31, 32]. In vitro studies have shown that cells are located on two sides of a high viscosity gelatinous liquid environment without movement to each other [33]. In this study, we increased the viscosity (3%) of glycerol to prolong the absorption time and to reveal a longer mechanical separation effect.

The positive effects of various boron compounds on the wound healing process have been demonstrated before [10–12]. In in-vitro and in-vivo studies performed by our group, sodium pentaborate has been shown to exert anti-inflammatory effects through cell proliferation, cell migration, and growth factor expression pathways and to accelerate wound healing in different wound models [12, 13]. In a prospective randomized clinical trial, we also revealed that sodium pentaborate gel prevents radiation induced dermatitis in breast cancer patients [14].

In this study, we observed that 3% sodium pentaborate or 3% glycerol did not prevent PPA formation when either was applied to the peritoneal surface. However, when we applied both molecules together, we created the synergistic effect and PPA was statistically reduced.

We conclude that according to the anti-inflammatory and wound healing acceleration activity of sodium pentaborate, less inflammation occurred and less adhesive molecules were produced with faster wound healing. Meanwhile, 3% dense glycerol ensured effective mechanical separation around the wound healing environment. This synergistic activity revealed less PPA formation.

The results of a macroscopically low PPA score with a high histopathologic fibrosis grade in the glycerol group and, in contrast, a macroscopically high PPA score with low histopathologic fibrosis grade in the sodium pentaborate group support our hypothesis.

In conclusion, 3% glycerol plus 3% sodium pentaborate as a new formulation has effective preventive effects on PPA by a synergistic mechanism. In order to use this new formulation in abdominal surgical interventions, its effects on different surgical procedures, especially anastomotic healing, should be evaluated.

## Figures and Tables

**Table 1:** Definitions of macroscopic adhesion scoring.

Grades	Adhesion size definiton	Adhesion severity definition
0	No adhesion	No adhesion
1	Adhesions cover a maximum of 1/3 of the model area	Spontaneously separating adhesion
2	Adhesions cover a maximum of 2/3 of the model area	Separation of adhesion with traction
3	Whole model area covered with adhesions	Separation of adhesion with sharp dissection

**Table 2:** Definitions of histopathologic fibrosis scoring.

Grades	Definition
0	No fibrosis (no fibroblasts and/or collagen fibers)
1	Slight fibrosis (few fibroblasts and/or collagen fibers)
2	Median fibrosis (more fibroblasts and/or collagen fibers)
3	Severe fibrosis (lots of fibroblasts and/or collagen fibers)

**Table 3:** Grades of macroscopic adhesion size.

Grade	0.9% NaCl group (*n*)	3% glycerol group (*n*)	3% sodium pentaborate group (*n*)	3% glycerol plus 3% sodium pentaborate group (*n*)
0	—	2	-	5
1	—	4	2	2
2	1	1	3	—
3	6	—	2	—
Total score	19	6	14	2

**Table 4:** Grades of macroscopic adhesion severity.

Grade	%0.9 NaCl group (*n*)	3% glycerol group (*n*)	3% sodium pentaborate group (*n*)	3% glycerol plus 3% sodium pentaborate group (*n*)
0	—	2	—	5
1	—	3	2	2
2	—	2	4	—
3	7	—	1	—
Total				
Score	21	7	13	2

**Table 5:** Grades of histopathologic fibrosis.

Grade	%0.9 NaCl group (*n*)	3% glycerol group (*n*)	3% sodium pentaborate group (*n*)	3% glycerol plus 3% sodium pentaborate group (*n*)
0	—	2	—	5
1	—	2	5	2
2	2	1	1	—
3	5	2	1	—
Total score	17	10	10	1

## Data Availability

The data used to support the findings of this study are included within the article.
